# Visually Evoked Visual-Auditory Changes Associated with Auditory Performance in Children with Cochlear Implants

**DOI:** 10.3389/fnhum.2017.00510

**Published:** 2017-10-24

**Authors:** Maojin Liang, Junpeng Zhang, Jiahao Liu, Yuebo Chen, Yuexin Cai, Xianjun Wang, Junbo Wang, Xueyuan Zhang, Suijun Chen, Xianghui Li, Ling Chen, Yiqing Zheng

**Affiliations:** ^1^Department of Otolaryngology, Sun Yat-sen Memorial Hospital, Sun Yat-sen University, Guangzhou, China; ^2^Department of Hearing and Speech Science, Xin Hua College of Sun Yat-sen University, Guangzhou, China; ^3^Department of Biomedical Information Engineering, School of Electrical Engineering and Information, Sichuan University, Chengdu, China; ^4^Department of Clinical Medicine, Sun Yat-sen University, Guangzhou, China

**Keywords:** cochlear implant, cross-modal reorganization, sLORETA, visual compensation

## Abstract

Activation of the auditory cortex by visual stimuli has been reported in deaf children. In cochlear implant (CI) patients, a residual, more intense cortical activation in the frontotemporal areas in response to photo stimuli was found to be positively associated with poor auditory performance. Our study aimed to investigate the mechanism by which visual processing in CI users activates the auditory-associated cortex during the period after cochlear implantation as well as its relation to CI outcomes. Twenty prelingually deaf children with CI were recruited. Ten children were good CI performers (GCP) and ten were poor (PCP). Ten age- and sex- matched normal-hearing children were recruited as controls, and visual evoked potentials (VEPs) were recorded. The characteristics of the right frontotemporal N1 component were analyzed. In the prelingually deaf children, higher N1 amplitude was observed compared to normal controls. While the GCP group showed significant decreases in N1 amplitude, and source analysis showed the most significant decrease in brain activity was observed in the primary visual cortex (PVC), with a downward trend in the primary auditory cortex (PAC) activity, but these did not occur in the PCP group. Meanwhile, higher PVC activation (comparing to controls) before CI use (0M) and a significant decrease in source energy after CI use were found to be related to good CI outcomes. In the GCP group, source energy decreased in the visual-auditory cortex with CI use. However, no significant cerebral hemispheric dominance was found. We supposed that intra- or cross-modal reorganization and higher PVC activation in prelingually deaf children may reflect a stronger potential ability of cortical plasticity. Brain activity evolution appears to be related to CI auditory outcomes.

## Introduction

Over the past several decades, individuals with severe to profound sensorineural hearing loss mostly benefitted from cochlear implants (CI). Individuals with CI can benefit from awareness of environmental sounds (Liang et al., 2014), better quality of life (Damen et al., 2007; Klop et al., 2007), and significant improvements in auditory speech perception (Lazard et al., 2010). However, evidence suggests that there is variability in auditory speech perception abilities among CI recipients (Blamey et al., 2013).

Many factors are associated with the auditory speech perception in CI recipients, including the age at which the CI was received, cognitive abilities, family environment, etiology, and speech-language therapy (Schramm et al., 2002; Liang et al., 2014; Sharma et al., 2015). Of these factors, age at implantation is the most important factor in terms of CI outcome (Sharma et al., 2015). For example, younger children with CI would achieve better speech outcomes. However, a large portion of variability in CI outcome remains unexplained by these models (Lazard et al., 2013). Such as in the study by Schramm et al. (2002), although their results showed CI patients with prelingual deafness achieved significantly better speech understanding using phonetically balanced monosyllabic words, there was a wide performance range among patients(Schramm et al., 2002; Lazard et al., 2013). They found that some older prelingually deaf children with CI also performed well in speech communication (Schramm et al., 2002). They suggested that this may be due to the extent of visual cross-modal impact on the auditory cortex.

Cross-modal reorganization has been reported in both blind and deaf individuals (Kujala et al., 2000; Finney et al., 2001; Fine et al., 2005; Sadato et al., 2005; Doucet et al., 2006). For example, neuroimaging studies using functional magnetic resonance imaging (fMRI) and magnetoencephalography (MEG) revealed that visual stimuli such as a moving dot pattern can activate certain regions of the auditory cortex (Brodmann’s areas 42 and 22) in prelingually deaf participants (Finney et al., 2001; Bavelier and Neville, 2002, 2003). In addition, some event-related potential (ERP) studies found larger ERP amplitudes and a greater anterior distribution of N1 components in deaf individuals when they processed the visual stimulus of an isoluminant color change (Armstrong et al., 2002). The evidence obtained from animal research in ferrets proves that auditory cortical function is weakened by cross-modal invasion (Mao et al., 2011; Mao and Pallas, 2013). The presence of a visual-auditory modality in early life offers opportunities for changes in individual behavior and audiological rehabilitation (Bavelier et al., 2006).

Visual cross-modal effects on the auditory cortex have been reported to play a role in CI outcomes (Lee et al., 2007a,b). For example, Lee et al. found hypometabolism in the temporal lobes of prelingually deaf children. Post-CI speech scores positively associated with enhanced metabolic activity in the prefrontal cortex, which contributes to auditory processing, and decreased metabolic activity in Heschle’s gyrus, which contributes to visual processing (Lee et al., 2007a). Sandmann et al. (2012) used parametrically modulated reverse checkerboard images to examine the initial stages of visual processing and confirmed visual take-over in the auditory cortex of CI recipients. In addition, the extent of visual processing in auditory cortices in postlingually deaf subjects was negatively related to CI outcomes (Sandmann et al., 2012). However, due to uncertainty in the status of the visual cross-modal impact on the auditory cortex, the effectiveness of CI outcomes is unlikely to be predicted for CI candidates, particularly for prelingually deaf children. However, a recent review suggested that focusing on the visual take-over of the auditory cortex may be too limited (Stropahl et al., 2017), and a cortex of multi-sensory processing may contribute to the CI outcomes (Kang et al., 2004).

Recently, visually evoked potentials (VEPs) have been used to investigate visual-auditory cross-modality in CI patients. Visually evoked frontotemporal N1 responses were reported to be related to visual processing in the auditory cortex (Buckley and Tobey, 2011; Bottari et al., 2014; Stropahl et al., 2016). Buckley and Tobey (2011) reported that in post-lingually deaf subjects, the higher N1 VEP responses in the right temporal lobe in children who had received a CI was related to poor speech perception. Moreover, different visual stimuli or ‘sound’ vs. ‘non-sound’ photos have been reported to produce different N1 responses in the frontotemporal area; ‘sound’ photo stimuli evoked stronger N1 responses than ‘non-sound’ photo stimuli (Proverbio et al., 2011). Our preliminary studies have shown that N1 amplitudes (especially on the right side) were strongest in the deaf children followed by those with poorly performing CIs, controls, and those with well-performing CIs (Liang et al., 2017; Liu et al., 2017). Our results indicate enhanced visual recruitment of the auditory cortices in prelingually deaf children. Additionally, the decrement in visual recruitment of auditory cortices was related to good CI outcome. However, the mechanism by which the visual impact on the auditory cortex occurs during the first year is still unclear.

The present study aimed to investigate visually evoked visual-auditory neural changes during the first-year follow-up of CI patients, to investigate the neural changes at different periods post-CI, and also to investigate the impact of visual-auditory neural changes on CI outcomes.

## Materials and Methods

### Participants

Twenty follow-up prelingually deaf children fitted with CIs on their right sides and worn for at least a year were recruited. The CIs fitted in the patients included several different types: (1) 10 MEDEL SONATAti100; three Cochlear Freedom (CI24RE); and seven Advanced Bionics (AB) HiRes 120. On the basis of the category of auditory performance (CAP) scores (Archbold et al., 1995) and according to the 12M follow-up, they were divided into two group: (1) 10 subjects (four boys and six girls, mean age 5.1 ± 0.90 years old, ranging from 4 to 6 years old) with CAP scores > 5 were assigned to the CI good CI performer group (GCP) and the remaining 10 (four boys and six girls, mean age 5.4 ± 1.0 years old, ranging from 4 to 6 years old) with CAP scores ≤ 5 were in the poor CI performer group (PCP) (Liang et al., 2014). All the patients underwent follow-ups with VEP at the time before CI use (0M) and 3M–12M (at 3 month intervals) post-CI. Ten age- and sex- matched normal-hearing children were recruited as the control group. **Table 1** provides detailed demographic information in conjunction with the communication mode (such as using sign language or oral communication) and socio-economic status.

**Table 1:** Detailed demographic information of the CI participants.

Group	Subject No.	Age range at implantation (Year)	Age range at diagnosis (year)	Education setting	Communication mode	Socioeconomic status	CAP scores
**Poor CI Performers**	
	001	3–4	At birth	Special school	Sign language	Middle class	3
	002	3–4	At birth	Special school	Sign language	Middle class	2
	003	4–5	1–2	Special school	Simple speech and Sign language	High class	4
	004	2–3	2–3	Special school	Simple speech + Sign language	Low class	4
	005	4–5	At birth	Special school	Simple speech + Sign language	Middle class	3
	006	2–3	1–2	Special school	Sign language	Middle class	5
	007	4–5	1–2	Special school	Sign language	High class	3
	008	4–5	2–3	Special school	Sign language	High class	2
	009	2–3	1–2	Special school	Simple speech + Sign language	Low class	3
	010	2–3	At birth	Special school	Simple speech + Sign language	Low class	3
**Good CI Performers**	
	101	5–6	At birth	Special school	Simple speech + Sign language	Low class	6
	102	3–4	At birth	Special school	Simple speech + Sign language	Low class	7
	103	2–3	1–2	Special school	Simple speech + Sign language	Middle class	7
	104	5–6	2–3	Special school	Simple speech + Sign language	High class	7
	105	3–4	At birth	Special school	Simple speech + Sign language	High class	8
	106	2–3	At birth	Special school	Simple speech + Sign language	Middle class	7
	107	4–5	At birth	Special school	Simple speech + Sign language	Middle class	6
	108	2–3	1–2	Special school	Simple speech + Sign language	Middle class	7
	109	2–3	At birth	Special school	Simple speech + Sign language	High class	7
	110	3–4	At birth	Special school	Simple speech + Sign language	Middle class	8

Ethical approval was obtained from the Institutional Review Board at Sun Yat-sen Memorial Hospital at Sun Yat-sen University. Detailed information was provided to the parents and parental written consent was obtained before proceeding with the study.

### Visual Stimuli

**FIGURE 1:**
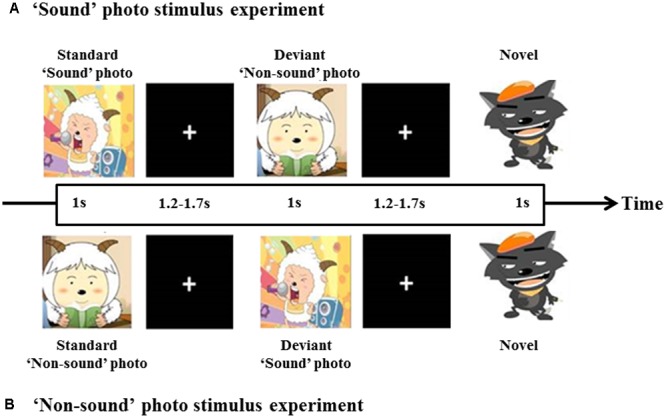
Diagram of the experimental block design. A total of 100 stimuli, 85% for standard and 15% for deviant. At least one standard stimulus was presented before each deviant stimulus was given. ‘Sound’ photo **(A)** and ‘Non-sound’ photo **(B)** stimulus experiment.

One ‘sound’ photo (a photograph with imaginative sound) and one ‘non-sound’ photo (a photograph without imaginative sound) were presented as visual stimuli in a manner similar to that in a study by Proverbio et al. (2011). The photographs were chosen to ensure that most of the children were familiar with the images and understood their meaning. **Figure 1** shows the experimental block design, a pseudo randomization sequence was used, which consisted of an intermittent stimulus mode using ‘sound’ and ‘non-sound’ photo stimuli. The ‘sound’ photo stimulus experiment consisted of 85 trials of ‘sound’ photo stimuli and 15 trials of ‘non-sound’ photo stimuli as deviant stimuli. In contrast, the ‘non-sound’ photo stimulus experiment consisted of 85 trials of ‘non-sound’ photo stimuli and 15 trials of ‘sound’ photo stimuli as deviant stimuli. As shown in **Figure 1**, each stimulus was presented for 1 s, followed by one blank screen (1.7–1.9 s in duration) as the inter-stimulus. To make sure that the participants concentrated on the stimuli, one novel stimulus that consisted of 15 photographs was presented after 5–10 trials, and the children were asked to press a button while the deviant photograph was present.

### Electroencephalography (EEG) Recordings

Event-related potentials (ERPs) were recorded from 128 scalp electrodes (Dense Array EEG System with HydroCel Geodesic Sensor Nets; EGI, Eugene, OR, United States). After installation of the 128-channel electrophysiological cap, testing occurred in a soundproof and electrically shielded room. Each participant was asked to sit on a comfortable chair approximately 100 cm away from the 19-inch high-resolution video graphics array (VGA) computer screen on which the visual stimuli were presented. All the photos were presented in the center of the screen, and the visual angle of the stimuli is zero degree. The participants were instructed to watch the screen throughout the entire experiment and avoid/minimize body and eye movements. The impedance for each electrode was kept below 40 kΩ during the experiment (Liang et al., 2014).

### EEG Data Analysis

ERP responses were recorded continuously using Net Station 4.3 (EGI, United States) and analyzed off-line. The ERP signals were digitally filtered with a band-pass of 0.1–30 Hz and signals with a segment of 700 ms, including 100 ms of pre-stimulus baseline. Any signal with an electro-oculography amplitude exceeding 75 μV was excluded as an artifact likely caused by eye movements or eye blinks. An amplitude exceeding 75 μV at any electrode site was defined as a poor channel. If there were ≥6 or more poor channels in a segment, this segment was considered a bad segment and then excluded. If <6 poor channels were present, the segment was considered valid, and each poor channel was replaced with the average value obtained from its surrounding channels. The response waveforms evoked by the visual stimuli were obtained by averaging all valid segments. All responses from individual electrodes were referred to Montage reference (Jung et al., 2006). The baseline was corrected according to the mean amplitude over the 100-ms pre-stimulus level.

All responses evoked by using either the ‘sound’ photo or ‘non-sound’ photo stimuli were recorded and averaged, respectively. The small-group average regions of interest were also analyzed. The N1 (the first negative response) FC4 (the right frontal-temporal area) to ‘sound’ photo were analyzed.

### Source Analysis

Comparison of latency and amplitude of ERP is sensor level-based. To further investigate cortical evolution of CI users and compare cortical activity differences between CI users and controls, we also conducted a source level activation analysis. Data analysis was performed with Brainstorm, which is documented and freely available for download online under the GNU general public license^1^ (Tadel et al., 2011). Brainstorm integrates various distributed source model methods and can readily conduct source analyses. We used Brainstorm to estimate primary auditory cortex (PAC), primary visual cortex (PVC), and parietal lobe cortex (PLC) activation. PAC and PVC have been described in the literature as involving cross modal reorganization by deaf children since these two parameters are directly related to the two most important sensations for deaf children. In fact, the parietal lobe, as the visual-auditory association cortex, integrates sensory information among various modalities and may also play a crucial role for deaf children in order to obtain auditory language ability. Several areas of the parietal lobe are important in language processing. Portions of the parietal lobe are involved in visuospatial processing (Pascual-Marqui, 2002). Therefore, in this study we also analyzed its activation pre- and post-CI. We applied the method of standardized low-resolution electromagnetic tomography analysis (sLORETA) to identify and evaluate active sources (Pascual-Marqui, 2002). The sLORETA method is based on a minimum norm estimation (MNE) and the activity (current density) is normalized by an individual estimate of the source standard deviation at each location. It has been identified as an efficient tool for functional mapping since it is consistent with physiology and capable of correct localization. The default anatomy ICBM152^2^ in Brainstorm was used for all the subjects as the template brain. The brain template was generated from the non-linear average of 152 subjects. Cortical surface was divided into 15,002 grid points (sources). In our study, when source current density was calculated, source orientation was constrained and was perpendicular to the cortical surface. In a first step, sLORETA source estimation was calculated based on 128-channel ERP data re-referenced to a common average. Secondly, based on our hypotheses, regions of interest (ROI) for primary visual and auditory areas and the parietal cortex were defined prior to statistical computations. The average source activation between duration 130–180 ms (exactly covering the N1 component for the ‘sound’ condition) in these ROIs were statistically compared between CI users and controls. These ROIs were defined based on the Mindboggle-atlas implemented in Brainstorm, which relies on automatic parcellation using a surface-based alignment of the cortical folding (Klein and Hirsch, 2005). PVC encompassed the lingual gyrus and pericalcarine sulcus (in Mindboggle atlas: ‘lingual L/R’ and ‘pericalcarine L/R’ approximately make up of PVC). Regarding PAC, previous studies have reported cross-modal activity in Brodmann areas 41 and 42 of deaf individuals and CI users (Bottari et al., 2014). Similarly, we defined the PAC as a small region in order to get a close approximation to Brodmann areas 41 and 42 (Mindboggle: transverse temporal L/R). PLC was defined as a combination of three small regions (Mindboggle: ‘inferiorparietal L/R,’ ‘precuneus L/R,’ ‘superiorparietal L/R,’ ‘supramarginal L/R’). The locations of the three ROIs on the cortex were outlined in **Figure 4** using red solid lines; PAC was indicated in **Figure 4A**, PLC in **Figure 4B**, and PVC in **Figure 4C**. Consistent with the ERP analysis, the source analysis focused on the time window of the N1 component. The individual absolute peak magnitude of the ROI source activation was defined as the average of a 50 ms window around the peak and was subjected to statistical analyses. Where appropriate, two-sample *t*-tests between the groups (deaf children with CI and controls) were applied for each ROI and each time duration after CI. In addition, two-sample *t*-tests between CI users with good outcome and those with poor outcome were also applied for each ROI and each time duration after CI.

### Statistical Analysis

One way analysis of variance (ANOVA) was used to compare the N1 amplitude and latency among the CI patients at 0M and 12M and in the controls. A two-way repeated-measures ANOVA was performed for the ERP data analysis. The within-subject factors were the follow-up months and the between-subject factor was the groups (GCP and PCP). The *post hoc* Tukey’s test was also used for multiple comparisons.

## Results

### Event Potential Component Analysis

**FIGURE 2:**
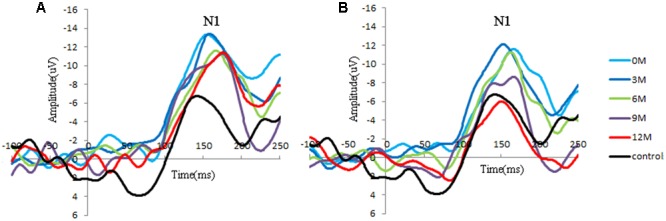
Group-averaged cortical changes of N1 components in CI children and controls. **(A)** Poor CI performers (PCP) and controls; **(B)** good CI performers (GCP) and controls. N1 is a negative response around 150 ms post-stimuli, and the peak amplitude is determined by the most negative point around 130–170 ms for each participant.

Distinct VEP morphological patterns were observed in all subjects, including the controls and CI children (i.e., **Figure 2**).

N1 amplitude and latency at FC4 for the GCP and PCP groups were analyzed. One way ANOVA was used to compared the N1 amplitude and latency among the CI patients at 0M and 12M and in controls. A two-way RM-ANOVA was used with one between-subject factor (groups: PCP and GCP) and one within-subject factor (months: 0M–12M measured at 3 month intervals).

Significant differences in N1 amplitude was found among GCP, PCP, and controls (*F* = 8.894, *p* = 0.000). And PCP had higher N1 amplitude (-12.9 μV) than GCP (-11.3 μV, LSD: *p* < 0.001) as well as controls (-10.6 μV, *p* = 0.002). However, no statistically significant differences was found between GCP and controls (*p* = 0.298).

While comparing the N1 amplitudes across the months and between groups, major significant effects were obtained for group (*F* = 23.347, *p* < 0.001) and months (*F* = 27.119, *p* < 0.001) (**Figure 2**). Group^∗^ month interaction effects were also found to be significant (*F* = 4.734, *p* = 0.002). Further simple effect test showed that for GCP, the N1 amplitude significantly increased at 3M [LSD: 3M (-14.8 μV) vs. 0M (-11.0 μV), *p* < 0.001], then decreased at 6M post-CI [6M (-10.0 μV) vs. 3M, *p* < 0.001], but increased again at 9M [6M vs. 9M (-11.0 μV), *p* = 0.048]. However, there were no significant difference between 9M and 12M (-10.1 μV) post-CI (*p* = 0.145). For PCP, N1 amplitude showed no significant difference between 0M (-14.3 μV) and 3M (-14.3 μV), then decreased at 6M (-11.3 μV) post-CI (6M vs. 3M, *p* < 0.001), but increased again at 9M (-13.1 μV) (6M vs. 9M, *p* = 0.006), and finally decreased at 12M (-11.3 μV) (9M and 12M, *p* = 0.006) (**Table 2**).

**Table 2A:** The N1 amplitude across the groups.

	GCP	PCP	Controls	*p*
0M(μV)	–11.0 ± 1.23	–14.3 ± 2.57	–	<0.001
3M	–14.8 ± 1.39	–14.3 ± 1.52	–	ns
6M	–9.74 ± 1.02	–11.3 ± 0.88	–	0.018
9M	–11.0 ± 1.04	–13.1 ± 2.02	–	0.002
12M	–10.1 ± 1.06	–11.3 ± 0.79	–10.6 ± 1.62	<0.001
*ns, No statistical significance.*				

**Table 2B:** The N1 latency across the groups.

	GCP	PCP	Controls
0M(ms)	162.5 ± 17.59	138.6 ± 10.32	–
3M	159.7 ± 15.94	151.8 ± 8.92	–
6M	163.3 ± 17.48	171.3 ± 20.25	–
9M	146.3 ± 9.53	143.7 ± 13.03	–
12M	149.2 ± 8.53	167.7 ± 10.28	150.3 ± 10.50

Through simple effect test, we compared N1 component in each measured month. For amplitude, at 0M, 6M, and 9M GCP showed significant difference against PCP (0M: *p* < 0.001; 6M: *p* < 0.001; 9M: *p* = 0.002). Precisely speaking, whether in 0M, 6M, and 9M, N1 Amplitude in GCP were lower than that in PCP (0M: GCP and PCP = -11.0 μV and -14.3 μV; 6M: GCP and PCP = -9.7 μV and -11.3 μV; 9M: GCP and PCP = -11.0 μV and -13.1 μV).

For N1 latency, there were no major, significant effects for groups (*F* = 0.329, *p* = 0.574), months (*F* = 0.449, *p* = 0.511).

### Source Analysis

**FIGURE 3:**
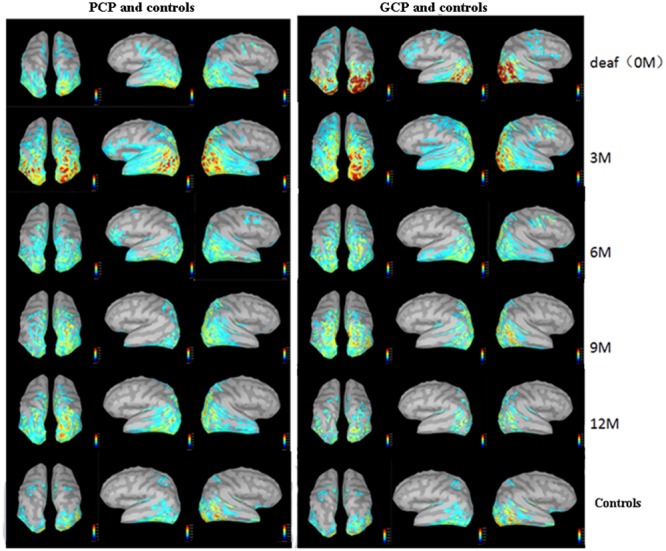
Temporal evolutions of group-averaged cortical changes of the N1 component in deaf children pre-and post-CI and cortical activation in controls. The **(left)** part shows cortical-activated evolution of PCP and controls, while the **(right)** one shows those of GCP and controls. The last row shows cortical activation in normal controls.

The source energy was analyzed according to the N1 response (130–180 ms). **Figure 3** demonstrate temporal dynamical evolutions of cortical changes from 0M (deaf) to 12M on group-averaged N1 cortical responses of GCP, PCP (GCP) and PCP. Obviously, both GCP and PCP had stronger responses in PAC, PVC, and PLC than the controls. The energy decrease was significant in the occipital area (including PVC) in GCP post-CI, which was close to level of the controls at 12M. For PCP, the energy at the occipital lobe slightly increased at 3M post-CI and decreased as the CI was used, but the source energy was still higher than the controls. For the temporal area, both GCP and PCP had slight energy source increases at 3M which was followed by a decrease. While the good performers were similar to the control, the differences were still significant on the right side.

**FIGURE 4:**
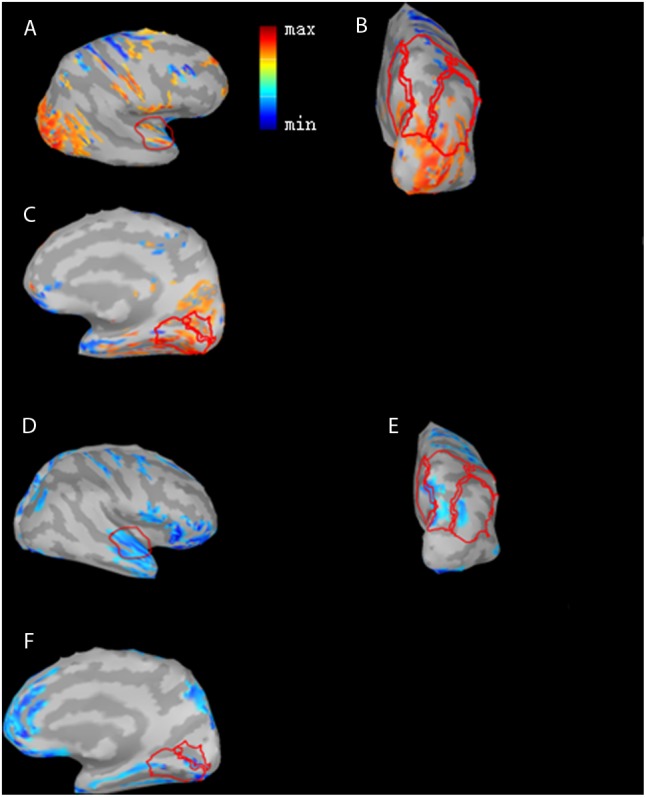
Differences in N1 cortical responses between GCP and PCP at 0 month (0M) and the 12th month (12M). Red color indicates GCP > PCP (PAC **(A)**, PLC **(B)**, and PVC **(C)**) at 0M while blue indicates PCP > GCP at 12M (PAC **(D)**, PLC **(E)** and PVC **(F)**). The activation differences of the three ROI cortical regions, PAC **(A)**, PLC **(B)**, and PVC **(C)**, and GCP were significantly larger than PCP for the three ROIs (*p* = 0.00538, *t* = 3.30 for PAC, *p* = 4.70e-06, *t* = 7.18 for PVC, and *p* = 0.0233, *t* = 2.55 for PLC) at 0M and descriptively smaller than PCP at 12M.

**Figure 4** shows differences in N1 cortical activation between GCP and PCP at 0M and 12M. Three ROIs, the PAC (**Figures 4A,D**), PLC (**Figures 4B,E**), and PVC (**Figures 4C,F**) were outlined with a red line. The three ROIs for GCP were significantly larger than those of PCP (*t* = 2.55, *p* = 0.023 for PLC, *t* = 7.18, *p* = 0.012, for PVC, *t* = 3.30 *p* = 0.005, for PAC) at 0M. At 12M, the ROI for the GCP was smaller than for the PCP groups, suggesting that PAC (*t* = -1.98, *p* = 0.071) of PCP had more residual take-over of the auditory cortex. Compared to GCP at 12M, PCP requires more energy and cognitive resources for low (mainly PVC; *p* = 0.507), moderate/high levels of visual processing (mainly PLC; *p* = 0.704).

**FIGURE 5:**
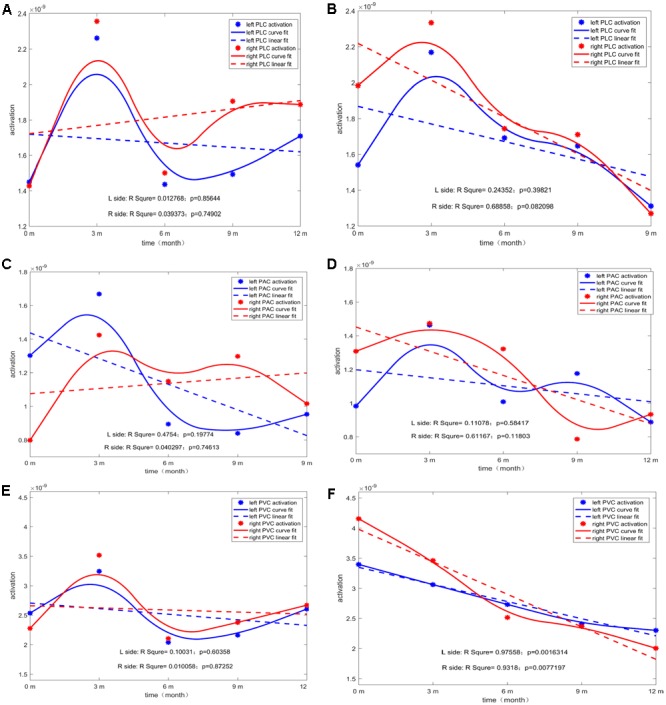
The temporal evolution of average activation of the two ROIs (left and right) as a function of the duration of CI for both GCP and PCP. Evolution of left or right PLC with the increase in CI use for PCP **(A)** and GCP **(B)**. Evolution of left or right PAC with the increase in CI use for PCP **(C)** and GCP **(D)**. Evolution of left or right PVC with the increase in CI use for PCP **(E)** and GCP **(F)**.

For each type of ROI located at the left or right hemisphere, two types of curve fitting methods were used: (1) Type 1: MATLAB function spcrv was used, which can conduct uniform B-spline curve fitting of order 3 and 2.) Type 2: MATLAB function regression was used to conduct linear regression of average ROI activation over five stages (0M–12M at 3 months intervals). **Figure 5** shows the evolution of the average activation of three ROIs as a function of the duration of CI experience. For PCP, with increasing CI experience, PVC slightly increased at 0M and then decreased followed by a final increase up to the same level as that at 0M. For GCP, PVC significantly and linearly decreased to a lower level. For both left and right PVC, the p value was small (left *p* = 0.002; right *p* = 0.007), suggesting that when comparing the source change in deaf children between GCP and PCP groups, the manner in which PVC activation evolves from 0M to 3M is a sensitive precursor for differentiating between GCP and PCP. As shown in **Figure 3**, for PCP, the energy at the occipital areas slightly increased in the first month post-CI, and then decreased as the CI was used. At the occipitotemporal area, similar changes were seen.

## Discussion

This study investigated photo processing in prelingually deaf CI children and what occurs in visual central processing as a result of the CI-induced auditory-derived experience. Unfortunately, CI children are unable to undergo functional MRI or positron emission computed cosmography. Other technologies that are functional near infrared spectroscopy may not always be fully exploited in CI research (Chen et al., 2015; Lawler et al., 2015). Therefore, our present study used ERP components and distributed source localization originating from risk-free high-density EEG recordings to carefully identify and follow cortical activity in CI users. Compared to controls, the PCP group showed significantly larger N1 amplitudes; these results add to the evidence that stronger visual processing in the auditory areas that are negatively related to poor CI outcome also occur in prelingually deaf children. However, the source analysis showed that good CI outcomes were associated with stronger auditory cortex activation by visual stimuli and significant decrements over the 12-month follow-up period. Moreover, the occipital energy source analysis suggested a linear decrement in activation that was associated with better CI outcomes. Increments in the visual-auditory cortex were also related to the better CI outcomes. As expected, visual activation decreases at the visual or auditory cortices were both related to better CI outcomes.

### Cross-Modal Reorganization for Prelingually Deaf Children

At the scalp level, stronger N1 VEP amplitudes in response to visual stimuli in the right front-temporal area in prelingually deaf were detected, and at the source level we showed higher activation at occipital and temporal cortical sources. As the CI was progressively used, GCP demonstrated significant decreases in N1 amplitude. These results added to the view that auditory deprivation does not only change the function of auditory cortex, but also the visual cortex in deaf subjects, even in pre-lingually deaf children. This was consistent with the findings from a previous study that demonstrated that enhanced right temporal lobe brain responses were negatively related to speech perception in pre-lingually deaf CI users (Buckley and Tobey, 2011). According to a report from Näätänen and Winkler, responses reflect the initial access to mental auditory representation (Näätänen and Winkler, 1999; Proverbio et al., 2011). Recently, Campbell and Sharma (2016) demonstrated that the visually evoked N1 component in a CI patient exhibited evidence of activation of the right temporal area, including the auditory cortex. The result suggested the presence of visual cross-modal plasticity in deaf and pre-lingually deaf children. Other reports also demonstrated that larger N1 components were consistently related to visual cross-modal re-organization in profoundly deaf adults, CI adults, and adults with hearing loss (Neville and Lawson, 1987; Buckley and Tobey, 2011; Campbell and Sharma, 2014). In our present study, the higher activation of the N1 component at 0M post -CI and also the stronger activation of the auditory cortex suggested a pattern of visual cross-modal reorganization of the auditory cortex in pre-lingually deaf children. In addition, it has been suggested that the decreased in VEP N1 latency is also related to visual cross-modal re-organization (Campbell and Sharma, 2014; Kok et al., 2014; Clemo et al., 2016). However, the CI children at 0M demonstrated longer N1 latency. This might be due to the ‘sound’ photo used in the present study; the complex visual stimulus may call for more synaptic cooperation. As the CI was used, decreased NI latency in GCP and increased N1 latency in PCP also supported the idea of visual cross-modal re-organization in CI children.

### CI-Induced Brain Activation Evolution and CI Outcomes

With the use of the CI, the N1 amplitude decreased. This is in agreement with reports in which the reversal of visual take-over of auditory cortex post-CI (Sandmann et al., 2012; Strelnikov et al., 2013; Stropahl et al., 2016). Additionally, participants with well-performing CIs achieved a greater decrement in N1 VEP amplitudes than those with poorly performing CIs. The ERP pattern (strong N1 response in the frontal area and weak response in the occipital area) reported by Proverbio, which involved sound and non-sound photo stimuli, was different from the reports by Doucet and Buckley, who used visual motion stimuli (a strong N1 response in the occipital area and a weak response in the occipital area (Doucet et al., 2006; Buckley and Tobey, 2011; Proverbio et al., 2011). Our ERP pattern results were consistent with the findings of Proverbio (Proverbio et al., 2011). Considering the visual and implicit nature of our experiments in which study participants focused on novel stimuli and ignored other images, the N1 VEP response indicated automatic and early visual processing of objective sound properties and the photo with imagery sound (‘sound’ photo). Based on these findings, we assume that the decrease of the automatic processing of auditory information in the frontal areas could be attributed to better CI outcomes (Senkowski et al., 2007).

### Decreased Activity in Visual and Auditory Regions after CI

The most significant decrease in brain activity was following CI was observed in the PVC, especially in GCP (see **Figure 5F**). Also, for PAC, there was a decreased trend in GCP (see **Figure 5D**), but not in PCP. According to recent reports by Stropahl, intra-modal and cross-modal re-organization may occur in post-lingually deaf patients post-CI (Stropahl et al., 2015, 2016). As noted previously, visual cross-modal take-over has been demonstrated in postlingually deaf adults and visual take-over was related to the auditory performance of the patients after receiving a CI (Sandmann et al., 2012; Strelnikov et al., 2013). The adaptation process after a CI procedure may partly indicate a reversal of auditory functional take-over, and insufficient adaptation to the new input may be reflected by residual signs of visual take-over (Lee et al., 2001; Sandmann et al., 2012). Our results support the findings that in the prelingually deaf children, intra- and cross-modal re-organization evolution can affect CI outcomes.

Furthermore, in our study, we found higher PVC activation at 0M and a significant decrease in the source energy after the CI was used were related to CI outcomes. Previous studies showed that in post-lingually deaf adults, only the higher activation of the auditory cortex was found and was related to poor CI outcomes (Doucet et al., 2006; Sandmann et al., 2012; Strelnikov et al., 2013; Heimler et al., 2014). In our present study, the prelingually deaf children did not show significantly lower activation at the PAC. We assume that prelingually deaf children must face the situation of the lack of auditory function, and they must use their visual abilities to compensate for this. The higher activation in the PVC may reflect a stronger potential in cortical plasticity in these children. After they receive the CI, there is stronger potential in cortical plasticity making the cortex more prone to adapt to the stimuli from CI and achieve good outcomes. This was confirmed in our study in which the GCP group showed a significant decreasing trend in the primary visual and auditory cortices.

### Change of Activity in Visual-Auditory Cortex Post-CI

Another interesting finding from the present study occurred at the PLC (which is also considered the visual-auditory cortex) in which the source energy decreased as CI was used for the GCP but not for PCP group (see **Figures 5A,B**). Our study did not find statistically significant evidence that the brain-activated evolution in this case correlated with the CI outcome. The decreased activity in this region could be explained by a decrease of the visual cross-modal re-organization or decreased demand for a visual cue visual information the processing that might be caused by the an increase in new reorganization caused by the input from the CI.

### Cerebral Hemispheric Dominance

It has been shown that the left and right temporal lobes play different roles in processing auditory information. The right lobe mainly participates in speech perception tasks in subjects with normal hearing and varies according to the degree of residual hearing. Right temporal lobe structures can be recruited for speech perception processing if the speech signal is degraded and seems to be important for underlying meaning in message extraction (Meyer et al., 2002; Liikkanen et al., 2007). However, the left temporal lobe mainly processes fine structures of speech signals structures (Friederici and Alter, 2004). In addition, several studies involving deaf individuals and CI users have shown that the effects of deprivation-induced cross-modal plasticity have been primarily localized to the right hemisphere, either because the rig ht hemisphere is more susceptible to reorganizational changes compared with the left hemisphere or because the right hemisphere is more involved in the processing of sounds with low complexity (Finney et al., 2001; Hine and Debener, 2007; Buckley and Tobey, 2011; Rouger et al., 2012; Sandmann et al., 2012; Lazard et al., 2013; Lyness et al., 2013). However, our study did not show significant dominance on the right side (see **Figure 5**). This might be a due to the choice stimuli. It had been reported that when presented laterally, the N1 response was contralateral to the visual field of the stimulus (Wascher et al., 2009; Buckley and Tobey, 2011). Differences in the stimulus category of the two studies may be responsible the discrepancy between the two outcomes. Buckley used a vision motion stimulus in the peripheral visual field (Buckley and Tobey, 2011). In the present study, we presented the stimuli centrally, which produced bilateral temporal enhancement. However, in the PLC, slightly higher source energy was found on the right, and this might have occurred as a result of the use of ‘sound’ photos, which might call for the right side of the cortex to be involved in processing the meaning of the photo.

## Conclusion

The present ERP study investigated the photo processing of CI children during the follow-up period (12M). With regard to the CI use, GCP demonstrated significant decrement in N1 amplitude, and significant decrease in brain activity following CI was in PVC, also in PAC and GCP. Higher activation of the PVC at 0M and a significant decrease in source energy after the CI was used were related to the CI outcome. Our results revealed that intra- and cross-modal reorganization in prelingually deaf children had occurred, and higher activation of PVC may reflect a stronger potential in cortical plasticity. Brain activity evolution appeared to be related to the auditory performance of the CI children.

## Ethics Statement

This study was carried out in accordance with the recommendations of guidelines of Ethics Committee of Sun Yat-sen University with written informed consent from all subjects. All subjects gave written informed consent in accordance with the Declaration of Helsinki. The protocol was approved by the Ethics Committee of Sun Yat-sen University.

## Author Contributions

YZ designed the experiments and revised the manuscript; ML, JZ, and JL analyzed the data and wrote the manuscript; YBC, YXC, XW, JW, XZ, SC, XL, and LC help to improve the manuscript.

## Conflict of Interest Statement

The authors declare that the research was conducted in the absence of any commercial or financial relationships that could be construed as a potential conflict of interest.

## Footnotes

1 http://neuroimage.usc.edu/brainstorm

2 http://www.bic.mni.mcgill.ca/ServicesAtlases/ICBM152NLin2009

## References

[B1] Archbold S. Lutman M. E. Marshall D. H. (1995). Categories of auditory performance. *Ann. Otol. Rhinol. Laryngol. Suppl.* 166 312–314. 7668685

[B2] Armstrong B. A. Neville H. J. Hillyard S. A. Mitchell T. V. (2002). Auditory deprivation affects processing of motion, but not color. *Brain Res. Cogn. Brain Res.* 14 422–434. 10.1016/S0926-6410(02)00211-2 12421665

[B3] Bavelier D. Dye M. W. Hauser P. C. (2006). Do deaf individuals see better? *Trends Cogn. Sci.* 10 512–518. 17015029 10.1016/j.tics.2006.09.006 PMC2885708

[B4] Bavelier D. Neville H. J. (2002). Cross-modal plasticity: Where and how? *Nat. Rev. Neurosci.* 3 443–452. 12042879 10.1038/nrn848

[B5] Blamey P. Artieres F. Baskent D. Bergeron F. Beynon A. Burke E. (2013). Factors affecting auditory performance of postlinguistically deaf adults using cochlear implants: an update with 2251 patients. *Audiol. Neurootol.* 18 36–47. 10.1159/000343189 23095305

[B6] Bottari D. Heimler B. Caclin A. Dalmolin A. Giard M.-H. Pavani F. (2014). Visual change detection recruits auditory cortices in early deafness. *Neuroimage* 94 172–184. 10.1016/j.neuroimage.2014.02.031 24636881

[B7] Buckley K. A. Tobey E. A. (2011). Cross-modal plasticity and speech perception in pre- and postlingually deaf cochlear implant users. *Ear Hear.* 32 2–15. 10.1097/AUD.0b013e3181e8534c 20829699

[B8] Campbell J. Sharma A. (2014). Cross-modal re-organization in adults with early stage hearing loss. *PLOS ONE* 9:e90594. 10.1371/journal.pone.0090594 24587400 PMC3938766

[B9] Campbell J. Sharma A. (2016). Visual cross-modal re-organization in children with cochlear implants. *PLOS ONE* 11:e0147793. 10.1371/journal.pone.0147793 26807850 PMC4726603

[B10] Chen L. C. Sandmann P. Thorne J. D. Herrmann C. S. Debener S. (2015). Association of concurrent fNIRS and EEG signatures in response to auditory and visual stimuli. *Brain Topogr.* 28 710–725. 10.1007/s10548-015-0424-8 25589030

[B11] Clemo H. R. Lomber S. G. Meredith M. A. (2016). Synaptic basis for cross-modal plasticity: enhanced supragranular dendritic spine density in anterior ectosylvian auditory cortex of the early deaf cat. *Cereb. Cortex* 26 1365–1376. 10.1093/cercor/bhu225 25274986 PMC4785938

[B12] Damen G. W. Beynon A. J. Krabbe P. F. Mulder J. J. Mylanus E. A. (2007). Cochlear implantation and quality of life in postlingually deaf adults: long-term follow-up. *Otolaryngol. Head Neck Surg.* 136 597–604. 10.1016/j.otohns.2006.11.044 17418258

[B13] Doucet M. E. Bergeron F. Lassonde M. Ferron P. Lepore F. (2006). Cross-modal reorganization and speech perception in cochlear implant users. *Brain* 129 3376–3383. 10.1093/brain/awl264 17003067

[B14] Fine I. Finney E. M. Boynton G. M. Dobkins K. R. (2005). Comparing the effects of auditory deprivation and sign language within the auditory and visual cortex. *J. Cogn. Neurosci.* 17 1621–1637. 10.1162/089892905774597173 16269101

[B15] Finney E. M. Clementz B. A. Hickok G. Dobkins K. R. (2003). Visual stimuli activate auditory cortex in deaf subjects: evidence from MEG. *Neuroreport* 14 1425–1427. 10.1097/00001756-200308060-00004 12960757

[B16] Finney E. M. Fine I. Dobkins K. R. (2001). Visual stimuli activate auditory cortex in the deaf. *Nat. Neurosci.* 4 1171–1173. 10.1038/nn763 11704763

[B17] Friederici A. D. Alter K. (2004). Lateralization of auditory language functions: a dynamic dual pathway model. *Brain Lang.* 89 267–276. 10.1016/S0093-934X(03)00351-1 15068909

[B18] Heimler B. Weisz N. Collignon O. (2014). Revisiting the adaptive and maladaptive effects of crossmodal plasticity. *Neuroscience* 283 44–63. 10.1016/j.neuroscience.2014.08.003 25139761

[B19] Hine J. Debener S. (2007). Late auditory evoked potentials asymmetry revisited. *Clin. Neurophysiol.* 118 1274–1285. 10.1016/j.clinph.2007.03.012 17462945

[B20] Jung J. Morlet D. Mercier B. Confavreux C. Fischer C. (2006). Mismatch negativity (MMN) in multiple sclerosis: an event-related potentials study in 46 patients. *Clin. Neurophysiol.* 117 85–93. 10.1016/j.clinph.2005.09.013 16325469

[B21] Kang E. Lee D. S. Kang H. Lee J. S. Oh S. H. Lee M. C. (2004). Neural changes associated with speech learning in deaf children following cochlear implantation. *Neuroimage* 22 1173–1181. 10.1016/j.neuroimage.2004.02.036 15219589

[B22] Klein A. Hirsch J. (2005). Mindboggle: a scatterbrained approach to automate brain labeling. *Neuroimage* 24 261–280. 10.1016/j.neuroimage.2004.09.016 15627570

[B23] Klop W. M. Briaire J. J. Stiggelbout A. M. Frijns J. H. (2007). Cochlear implant outcomes and quality of life in adults with prelingual deafness. *Laryngoscope* 117 1982–1987. 10.1097/MLG.0b013e31812f56a6 17767086

[B24] Kok M. A. Chabot N. Lomber S. G. (2014). Cross-modal reorganization of cortical afferents to dorsal auditory cortex following early- and late-onset deafness. *J. Comp. Neurol.* 522 654–675. 10.1002/cne.23439 23897533

[B25] Kujala T. Alho K. Naatanen R. (2000). Cross-modal reorganization of human cortical functions. *Trends Neurosci.* 23 115–120. 10.1016/S0166-2236(99)01504-0 10675915

[B26] Lawler C. A. Wiggins I. M. Dewey R. S. Hartley D. E. (2015). The use of functional near-infrared spectroscopy for measuring cortical reorganisation in cochlear implant users: a possible predictor of variable speech outcomes? *Cochlear Implants Int.* 16(Suppl. 1) S30–S32. 10.1179/1467010014Z.000000000230 25614264

[B27] Lazard D. S. Bordure P. Lina-Granade G. Magnan J. Meller R. Meyer B. (2010). Speech perception performance for 100 post-lingually deaf adults fitted with Neurelec cochlear implants: comparison between Digisonic Convex and Digisonic SP devices after a 1-year follow-up. *Acta Otolaryngol.* 130 1267–1273. 10.3109/00016481003769972 20446821

[B28] Lazard D. S. Lee H. J. Truy E. Giraud A. L. (2013). Bilateral reorganization of posterior temporal cortices in post-lingual deafness and its relation to cochlear implant outcome. *Hum. Brain Mapp.* 34 1208–1219. 10.1002/hbm.21504 22287085 PMC6870107

[B29] Lee D. S. Lee J. S. Oh S. H. Kim S. K. Kim J. W. Chung J. K. (2001). Cross-modal plasticity and cochlear implants. *Nature* 409 149–150. 10.1038/35051653 11196628

[B30] Lee H. J. Giraud A. L. Kang E. Oh S. H. Kang H. Kim C. S. (2007a). Cortical activity at rest predicts cochlear implantation outcome. *Cereb. Cortex* 17 909–917. 16731883 10.1093/cercor/bhl001

[B31] Lee H. J. Truy E. Mamou G. Sappey-Marinier D. Giraud A. L. (2007b). Visual speech circuits in profound acquired deafness: a possible role for latent multimodal connectivity. *Brain* 130 2929–2941. 17906328 10.1093/brain/awm230

[B32] Liang M. Chen Y. Zhao F. Zhang J. Liu J. Zhang X. (2017). Visual processing recruits the auditory cortices in prelingually deaf children and influences cochlear implant outcomes. *Otol. Neurotol.* 38 1104–1111. 10.1097/MAO.0000000000001494 28727651

[B33] Liang M. Zhang X. Chen T. Zheng Y. Zhao F. Yang H. (2014). Evaluation of auditory cortical development in the early stages of post cochlear implantation using mismatch negativity measurement. *Otol. Neurotol.* 35 e7–e14. 10.1097/MAO.0000000000000181 24335940

[B34] Liikkanen L. A. Tiitinen H. Alku P. Leino S. Yrttiaho S. May P. J. (2007). The right-hemispheric auditory cortex in humans is sensitive to degraded speech sounds. *Neuroreport* 18 601–605. 10.1097/WNR.0b013e3280b07bde 17413665

[B35] Liu J. Liang M. Chen Y. Wang Y. Cai Y. Chen S. (2017). Visual cortex activation decrement following cochlear implantation in prelingual deafened children. *Int. J. Pediatr. Otorhinolaryngol.* 99 85–89. 10.1016/j.ijporl.2017.04.011 28688572

[B36] Lyness C. R. Woll B. Campbell R. Cardin V. (2013). How does visual language affect crossmodal plasticity and cochlear implant success? *Neurosci. Biobehav. Rev.* 37 2621–2630. 10.1016/j.neubiorev.2013.08.011 23999083 PMC3989033

[B37] Mao Y. T. Hua T. M. Pallas S. L. (2011). Competition and convergence between auditory and cross-modal visual inputs to primary auditory cortical areas. *J. Neurophysiol.* 105 1558–1573. 10.1152/jn.00407.2010 21273321 PMC3075293

[B38] Mao Y. T. Pallas S. L. (2013). Cross-modal plasticity results in increased inhibition in primary auditory cortical areas. *Neural Plast.* 2013:530651. 10.1155/2013/530651 24288625 PMC3833201

[B39] Meyer M. Alter K. Friederici A. D. Lohmann G. Von Cramon D. Y. (2002). FMRI reveals brain regions mediating slow prosodic modulations in spoken sentences. *Hum. Brain Mapp.* 17 73–88. 10.1002/hbm.10042 12353242 PMC6871847

[B40] Näätänen R. Winkler I. (1999). The concept of auditory stimulus representation in cognitive neuroscience. *Psychol. Bull.* 125 826–859. 10.1037/0033-2909.125.6.826 10589304

[B41] Neville H. J. Lawson D. (1987). Attention to central and peripheral visual space in a movement detection task. III. Separate effects of auditory deprivation and acquisition of a visual language. *Brain Res.* 405 284–294. 10.1016/0006-8993(87)90297-6 3567606

[B42] Pascual-Marqui R. D. (2002). Standardized low-resolution brain electromagnetic tomography (sLORETA): technical details. *Methods Find. Exp. Clin. Pharmacol.* 24(Suppl. D) 5–12. 12575463

[B43] Proverbio A. M. D’aniello G. E. Adorni R. Zani A. (2011). When a photograph can be heard: vision activates the auditory cortex within 110 ms. *Sci. Rep.* 1:54. 10.1038/srep00054 22355573 PMC3216541

[B44] Rouger J. Lagleyre S. Demonet J. F. Fraysse B. Deguine O. Barone P. (2012). Evolution of crossmodal reorganization of the voice area in cochlear-implanted deaf patients. *Hum. Brain Mapp.* 33 1929–1940. 10.1002/hbm.21331 21557388 PMC6870380

[B45] Sadato N. Okada T. Honda M. Matsuki K. Yoshida M. Kashikura K. (2005). Cross-modal integration and plastic changes revealed by lip movement, random-dot motion and sign languages in the hearing and deaf. *Cereb. Cortex* 15 1113–1122. 10.1093/cercor/bhh210 15563723

[B46] Sandmann P. Dillier N. Eichele T. Meyer M. Kegel A. Pascual-Marqui R. D. (2012). Visual activation of auditory cortex reflects maladaptive plasticity in cochlear implant users. *Brain* 135 555–568. 10.1093/brain/4awr329 22232592

[B47] Schramm D. Fitzpatrick E. Seguin C. (2002). Cochlear implantation for adolescents and adults with prelinguistic deafness. *Otol. Neurotol.* 23 698–703. 10.1097/00129492-200209000-00016 12218622

[B48] Senkowski D. Saint-Amour D. Kelly S. P. Foxe J. J. (2007). Multisensory processing of naturalistic objects in motion: a high-density electrical mapping and source estimation study. *Neuroimage* 36 877–888. 10.1016/j.neuroimage.2007.01.053 17481922

[B49] Sharma A. Campbell J. Cardon G. (2015). Developmental and cross-modal plasticity in deafness: evidence from the P1 and N1 event related potentials in cochlear implanted children. *Int. J. Psychophysiol.* 95 135–144. 10.1016/j.ijpsycho.2014.04.007 24780192 PMC4209331

[B50] Strelnikov K. Rouger J. Demonet J. F. Lagleyre S. Fraysse B. Deguine O. (2013). Visual activity predicts auditory recovery from deafness after adult cochlear implantation. *Brain* 136 3682–3695. 10.1093/brain/awt274 24136826

[B51] Stropahl M. Chen L. C. Debener S. (2016). Cortical reorganization in postlingually deaf cochlear implant users: intra-modal and cross-modal considerations. *Hear. Res.* 343 128–137. 10.1016/j.heares.2016.07.005 27473503

[B52] Stropahl M. Chen L. C. Debener S. (2017). Cortical reorganization in postlingually deaf cochlear implant users: intra-modal and cross-modal considerations. *Hear. Res.* 343 128–137. 10.1016/j.heares.2016.07.005 27473503

[B53] Stropahl M. Plotz K. Schonfeld R. Lenarz T. Sandmann P. Yovel G. (2015). Cross-modal reorganization in cochlear implant users: auditory cortex contributes to visual face processing. *Neuroimage* 121 159–170. 10.1016/j.neuroimage.2015.07.062 26220741

[B54] Tadel F. Baillet S. Mosher J. C. Pantazis D. Leahy R. M. (2011). Brainstorm: a user-friendly application for MEG/EEG analysis. *Comput. Intell. Neurosci.* 2011:879716. 10.1155/2011/879716 21584256 PMC3090754

[B55] Wascher E. Hoffmann S. Sanger J. Grosjean M. (2009). Visuo-spatial processing and the N1 component of the ERP. *Psychophysiology* 46 1270–1277. 10.1111/j.1469-8986.2009.00874.x 19744158

